#  Measuring and managing the work environment of the mid-level provider – the neglected human resource

**DOI:** 10.1186/1478-4491-7-13

**Published:** 2009-02-19

**Authors:** Eilish McAuliffe, Cameron Bowie, Ogenna Manafa, Fresier Maseko, Malcolm MacLachlan, David Hevey, Charles Normand, Maureen Chirwa

**Affiliations:** 1 Centre for Global Health, Trinity College, University of Dublin, Dublin, Ireland; 2 College of Medicine, University of Malawi, Blantyre, Malawi; 3 School of Psychology, Trinity College, University of Dublin, Dublin, Ireland

## Abstract

**Background:**

Much has been written in the past decade about the health workforce crisis that is crippling health service delivery in many middle-income and low-income countries. Countries having lost most of their highly qualified health care professionals to migration increasingly rely on mid-level providers as the mainstay for health services delivery. Mid-level providers are health workers who perform tasks conventionally associated with more highly trained and internationally mobile workers. Their training usually has lower entry requirements and is for shorter periods (usually two to four years). Our study aimed to explore a neglected but crucial aspect of human resources for health in Africa: the provision of a work environment that will promote motivation and performance of mid-level providers. This paper explores the work environment of mid-level providers in Malawi, and contributes to the validation of an instrument to measure the work environment of mid-level providers in low-income countries.

**Methods:**

Three districts were purposively sampled from each of the three geographical regions in Malawi. A total of 34 health facilities from the three districts were included in the study. All staff in each of the facilities were included in the sampling frame. A total of 153 staff members consented to be interviewed. Participants completed measures of perceptions of work environment, burnout and job satisfaction.

**Findings:**

The Healthcare Provider Work Index, derived through Principal Components Analysis and Rasch Analysis of our modification of an existing questionnaire, constituted four subscales, measuring: (1) levels of staffing and resources; (2) management support; (3) workplace relationships; and (4) control over practice. Multivariate analysis indicated that scores on the Work Index significantly predicted key variables concerning motivation and attrition such as emotional exhaustion, job satisfaction, satisfaction with the profession and plans to leave the current post within 12 months. Additionally, the findings show that mid-level medical staff (i.e. clinical officers and medical assistants) are significantly less satisfied than mid-level nurses (i.e. enrolled nurses) with their work environments, particularly their workplace relationships. They also experience significantly greater levels of dissatisfaction with their jobs and with their profession.

**Conclusion:**

The Healthcare Provider Work Index identifies factors salient to improving job satisfaction and work performance among mid-level cadres in resource-poor settings. The extent to which these results can be generalized beyond the current sample must be established. The poor motivational environment in which clinical officers and medical assistants work in comparison to that of nurses is of concern, as these staff members are increasingly being asked to take on leadership roles and greater levels of clinical responsibility. More research on mid-level providers is needed, as they are the mainstay of health service delivery in many low-income countries. This paper contributes to a methodology for exploring the work environment of mid-level providers in low-income countries and identifies several areas needing further research.

## Background

### Introduction

A health workforce crisis is crippling health service delivery in many low-income countries. High-income countries with high salaries and attractive living conditions are drawing qualified doctors and nurses from poorer countries to fill gaps in their own human resources pool. This migration of skilled labour is depleting human capital in many developing countries [1]. The human resource crisis in Malawi is acute. The country has one of the world's lowest doctor-patient ratios, with less than one doctor per 50 000 population, compared to WHO's Health for All recommended ratio of one doctor per 5000 patients [2]. In 2006 there were 266 doctors in Malawi serving a population of 12 million [3].

While there is clearly a need to scale up the health workforce in sub-Saharan Africa, the macroeconomic and fiscal reality that the region is facing present a significant challenge. Real GPD in the region is expected to grow at an average rate of 5.8% per year. As a result, salaries of additional staff may not be afforded [4]. One response to this has been to train lower-level staff who would command lower salaries [5]. Such a strategy has already been adopted by several countries that increasingly rely on mid-level cadres (such as medical assistants, clinical officers and registered nurses) to perform tasks normally assigned to doctors, and enrolled nurses performing tasks normally assigned to registered nurses to provide health care [6,7].

Dovlo's study indicated that Kenya, Malawi, Mozambique, Tanzania, Uganda and Zambia have such cadres who are doing essential medical tasks, especially in rural areas [8]. In Malawi, clinical officers are a major resource of the health sector; they give anaesthetics, provide medical care and undertake surgical procedures. Recent studies provide strong evidence for the clinical efficacy [9,10] and economic value [11] of mid-level cadres, particularly in the provision of emergency obstetric care. But for these professional groups to provide high-quality services it is important that they are suitably motivated and can be retained in the full range of health care settings. In order to develop strategies to improve the motivation and retention of these mid-level cadres, we must begin measuring and monitoring the key factors within their work environment that affect their performance.

The role of organizational attributes or the work environment is becoming increasingly important in ensuring that adequate staffing levels can be maintained in high-income countries, particularly in times of shortage [12]. Several studies have shown the link between these organizational attributes and job satisfaction [13-15], burnout [16], retention and recruitment [12,17], decreased mortality and healthier staff [15]. Little is known about the predictive value of these same organizational traits in low-income or resource-poor settings. This study aimed to understand the role of such attributes in the satisfaction, motivation and performance of mid-level providers in district health facilities in Malawi (a country with high vacancy rates for all staff cadres). It adapts and develops an instrument for assessing the motivational environment, applies it in rural areas in Malawi, and provides evidence of the factors that influence motivation, staff satisfaction and retention.

## Methods

The conceptual framework for this study is the Managing for Performance framework developed by the Joint Learning Initiative [4]. This framework identifies three workforce objectives – coverage, motivation and competence – to achieve health system performance. Most studies of mid-level cadres to date have addressed the competence objective. This study focuses on the workforce objective of motivation, a critical element in improving the efficiency and effectiveness of health system performance.

### Sample

Three districts were purposively sampled from each of the three administrative regions in Malawi. The study population includes all health professional workers in public, private and NGO health facilities in Thyolo, Dowa and Karonga districts. The sample consists of those who were willing to participate at the time the data collectors visited the facilities. Questionnaires were administered in 34 health facilities. In Karonga health district, one district hospital, two rural hospitals, six health centres (of which two were Christian Health Association of Malawi (CHAM) facilities), one private clinic and one NGO facility were visited. In Dowa, questionnaires were administered in three hospitals (two of which were CHAM), one rural hospital, three health centres and one private clinic. In Thyolo, two hospitals (one CHAM), one rural hospital and eight Ministry of Health (MoH) and four CHAM health centres were visited for the interviews. From a total of 374 health workers, 153 participated in the study, giving a response rate of 41%. Table 1 gives a breakdown of the job titles. Enrolled nurses, medical assistants and clinical officers and others are those cadres we refer to as mid-level. This cohort constitutes 86% of the population interviewed.

**Table 1:** Job titles of respondents

**Job Title**	**Frequency**	**Percentage**
Enrolled Nurse	78	52.0

Medical Assistant	35	23.3

Clinical Officer	13	8.7

Technician	10	6.7

Registered Nurse	8	5.3

Other	5	3.4

Medical Officer	1	0.7

Missing data	3	2

### Data Collection

Participants completed measures of perceptions of work environment, burnout, job satisfaction and promotion. Data collection used a questionnaire that was pilot-tested in two districts with 20 health workers of different cadres. Interviewees were asked to complete the questionnaire with the researcher present to provide guidance and clarification where necessary.

### Instruments

The Healthcare Providers Work Index (HPWI) is an adaptation of the Revised Nursing Work Index (NWI-R) developed by Aiken and her colleagues [18,19] from the Nursing Work Index (NWI) [16]. According to a review of the measurement of the Nursing Practice Environment [20], the original NWI was developed from a study of 39 American hospitals (known as the magnet hospitals) based on their reputations for good nursing care and their low vacancy and turnover rate during a nursing shortage [21,22]. The NWI-R differs from the NWI in that it focuses on the presence of organizational traits rather than nurse satisfaction and perceived productivity associated with these traits [19]. The initial NWI-R contained 55 of the original 65 NWI items. Further analysis led to the development of a shorter, 15-item version with items being categorized into three subscales; autonomy, control over the practice setting and nurse-physician relationships [17]. These scales have been used almost exclusively to measure the work environment of nursing staff [19]. In this study we have adapted the 15-item version for use with all health care providers.

#### Maslach's Burnout Inventory

The Maslach Burnout Inventory (MBI) is composed of three subscales measuring personal accomplishment, emotional exhaustion and depersonalisation (an unfeeling and impersonal response towards the recipients of one's care) [23]. Responses are given on a six-point scale, with higher scores for emotional exhaustion and depersonalization and lower scores for personal accomplishment representing greater burnout. The MBI is the gold-standard questionnaire in this area: it has been cited in more than 1000 studies and has previously been used with doctors and nurses in Africa, in particular in Malawi [24]. Strong reliability coefficients have been reported for each of the subscales in Africa [23].

Job satisfaction was explored through several items with scaled responses. None of the job satisfaction scales in the extant literature was entirely relevant and appropriate to the context of this research, as in addition to job satisfaction we also wished to explore intentions to leave and perceived likelihood of obtaining another position. The items were identified from (1) existing questionnaires, (2) a review of the relevant literature and (3) suggestions from a panel of researchers and policy-makers with expertise in the area. The questions were intended to be descriptive of the particular context of the research, not to be additive.

## Results

### Demographics of the study population

Of the total sample, 66 respondents were male (43.1%), 85 female (55.6%) and 2 did not state their gender. The majority of respondents were in full-time (144, 95.4%) and permanent (132, 87.4%) employment. Approximately one third (55, 37.4%) of the sample was aged 30 or younger, and the majority of the sample (114, 77.6%) was aged 50 or less. Table 1 gives a breakdown of job titles, the majority of respondents being enrolled nurses or medical assistants.

Combining the medical assistant and clinical officer grades gives a total of 48 (32%) mid-level medical cadres. Combining the nursing cadres gives a total of 86 (57.5%). Comparisons were made between these two groups (with the only medical officer – fully qualified doctor – in the sample being excluded from the analysis). The other nursing and medical cadres could all be described as mid-level providers, i.e. health workers who work beyond the level of responsibility usually afforded health workers with similar training in higher-income countries

The mean length of time spent working in the health service was 13.49 years, with the average length of the working week (over the past year) being 54.66 hours. More than one quarter of the respondents (39, 27.5%) did not belong to a professional organization.

### Principal components and Rasch analysis

Research with the NWI-R has reported differing factor structures, suggesting that there is variability in how the questionnaire items are understood across different samples. This may reflect the sensitivity of the questionnaire to the different contexts in which it has been used, or difficulties with particular items within the questionnaire. We therefore undertook two forms of analysis – a Principal Components Analysis to explore the factor structure, and a Rasch analysis to identify if the emergent factors were being optimally measured by the existing items. Rasch analysis on the HPWI identified one item with unacceptable fit: mean square infit = 2.26, mean square outfit = 2.15, standardized infit = 7.1, standardized outfit = 6.9 [25]. Following removal of the item, principal components analysis with varimax rotation was performed on the 14 items; four subscales, accounting for 59% of the variance in the items, were extracted (Table 2). The PCA with rotation was conducted to achieve simple structure in the data, with each item only loading on to a single factor and each factor determined by a number of strongly loading items [26,27]. Rotation of the extracted components produced a more interpretable solution than the unrotated solution.

**Table 2:** Factor loadings, variance explained and Cronbach's alpha reliability coefficients for the 14-item Health Care Providers Work Index.

**Subscale 1: Adequate resources (16.7%, α = .75)**	
.85	Enough staff to provide quality patient care

.77	Enough staff to get the work done

.64	Opportunity to work on a highly specialized patient care unit

.48	Enough time and opportunity to discuss patient care problems with other staff

**Subscale 2: Management support (16.3%, α = .76)**	

.80	A manager who is a good manager and leader

.74	A manager who backs up the staff in decision-making, even if the conflict is with a more qualified member of staff

.69	Hospital/clinic managers support and value health workers

**Subscale 3: Working relationships (14.4%, α = .65)**	

.44	Doctors, nurses and other health workers have good working relationships

.81	Collaboration (joint practice) between different cadres of health workers

.66	A lot of teamwork between the different cadres of health workers

.56	Adequate support services allow health workers to spend time with patients

**Subscale 4: Control over practice (11.8%, α = .54)**	

.74	Freedom to make important patient care and work decisions

.67	Patient care assignments that foster continuity of care, i.e. the same health workers care for the patient from one day to the next

.56	Health professionals control their own practice

**Figure 1:**
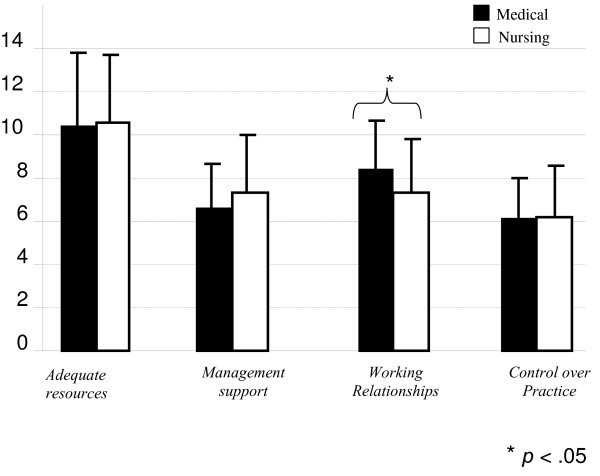
**Mean (SD) for medical and nursing staff on the work index**.

Figure 1 shows the mean scores on each of the four subscales for nursing and medical cadres. Inadequate resources and management support were most problematic in the work environments of these mid-level providers. The means also suggest less than full agreement on the presence of good working relationships and control over practice, but more staff members agree that these factors are present than the first two factors. Student's t-test revealed that medical cadres were significantly less likely (t(126) = 2.42, p < .05) than nursing staff to report the presence of positive working relationships (mean difference = 0.27, 95% CI = .05 to 0.50).

Scores on the burnout scale indicate that more than one third of the sample scored high on the emotional exhaustion scale (Table 3). This, coupled with the high percentage of the sample scoring low on personal accomplishment, indicates that burnout is a problem across the sample.

**Table 3:** Frequency (%) of sample in each category of the burnout subscales

**Burnout Subscale**	**Low**	**Moderate**	**High**
Emotional exhaustion	37 (34%)	39 (35%)	34 (31%)

Depersonalization	93 (77%)	22 (18%)	6 (5%)

Personal accomplishment	42 (45%)	26 (28%)	25 (27%)

Subscale 1 (Adequate resources) of the HPWI correlates positively (p < .05) with emotional exhaustion on MBI, i.e. those who believe that their work environments are inadequately resourced are more emotionally exhausted. Subscale 1 also correlates negatively with job satisfaction (Pearson r = -0.201; p < .05), satisfaction with profession (Pearson r = -0.277; p < .01), likelihood of leaving the job (Pearson r = -0.225; p < .01), and plans to leave the job within the next 12 months (t(138) = 3.38, *p *= .001, 95% CI 0.79 – 3.02). Two additional measures of job satisfaction – (a) actively seeking other employment (Pearson r = 0.228; p < 0.01) and (b) satisfaction with current job assignments (Pearson r = -0.361; p < 0.001) showed significant correlation with subscale 1.

Subscale 2 (Management support) correlated positively with actively seeking other employment (Pearson r = 0.243; p < .01) and negatively with satisfaction with current job assignments (Pearson r = -0.223; p < .01) i.e. the less perceived management support, the more likely staff were to report job dissatisfaction with current job assignments and the more likely they were to report actively seeking other employment.

Student's t-test revealed a significant (*t*(141) = 2.59, *p *< .05, 95%CI = 0.27 – 1.97) gender difference in responses to subscale 3 (Working relationships), with male workers reporting poorer working relationships in their work environments. Interestingly, a significant negative correlation Pearson r = -0.174; p < .05) arose with satisfaction with profession, satisfaction with current job assignments (r = -.0.291; p <.001) and with likelihood of leaving the job (Pearson r = -0.200; p < .05), but correlation with job satisfaction did not reach significance levels. Thus, in general reports of poor working relationships were associated with job dissatisfaction.

Subscale 4 (Control over practice) correlates negatively with satisfaction with salary/wages (Pearson r = -0.176; p < .05), and with current job assignments (r = -0.206; p < .05).

There was a strong negative correlation (Pearson r = -0.321, – 0.379, -0.373 and -0.294; all p < .01) between all four subscales of the HPWI and the number of years respondents had spent working in the health service, i.e. those who had longer work experience were more likely to perceive these (positive) organizational attributes as being present in their environment.

Comparisons using Student's t-tests between the medical and nursing mid-level cadres identified significant differences in terms of job satisfaction. Table 4 shows that nursing cadres were significantly more satisfied than medical cadres with their jobs and with their profession. Interestingly, nurses were more likely to indicate that they were thinking of leaving, but the groups did not differ in terms of actual plans to leave.

**Table 4:** Comparison of medical and nursing staff on job satisfaction items

	**Medical *M *(*SD*)**	**Nursing *M *(*SD*)**	**95%CI for Mean Difference**
On the whole, how satisfied are you with your job?	2.68 (1.0)	3.12 (0.9)*	**00.11 to 0.77**

Independent of your present job, how satisfied are you with your current profession?	2.58 (1.1)	3.07 (0.9)*	**0.14 to 0.84**

Thinking about the next 12 months, how likely do you think it is that you will choose to leave your present job?	2.46 (1.3)	2.95 (1.2)*	**0.05 to 0.94**

Do you plan to leave your present position?	2.46 (0.7)	2.60 (0.7)	**0.11 to 0.38**

* *p *< .05

Simultaneous multiple regression examined the contribution of the four HPWI subscales in accounting for variation in items relating to work satisfaction. While the multivariate four HPWI scales model accounted for 16% (*F*(4,147) = 6.79, *p *= .001) of the variation in satisfaction with current job assignments, only adequate resources made a significant independent contribution (t = -2.68, p = .008, partial r = .20) to the regression model. No other regression model was statistically significant.

## Discussion

The adaptation of the NWI-R has allowed us to develop a measure of work environment more broadly applicable to health workers. Previous studies that used the 15-item NWI-R scale with nursing cohorts have produced a variety of different subscales [17,19,11,28,29], with some replicating Aiken & Patrician's four-factor model and others identifying only three factors. Items (d), (e) and (h) in particular did not load onto any subscale in a number of previous studies [19,25,26]. Our analysis of the data from mid-level cadres has produced four distinct subscales: adequate resources, management support, work relationships and autonomy/control over practice accounting for almost 60% of the variance. With this cohort of health workers items, (d) "health professionals control their own practice", (e) "patient care assignments that foster continuity of care" and (h) "freedom to make important patient care and work decisions" load onto the factor autonomy/control over practice, which accounts for 8% of the total variance. In addition, item (g) "not being placed in a position of having to do things that are against my professional judgment" failed to load onto any factor and was therefore removed.

Scores on this revised Healthcare Providers Work-Index indicate that mid-level providers' work environments are particularly poor in terms of perceptions of resource adequacy, staff members indicating that they had neither sufficient staff nor time to do their work. Inadequate management support and a sense of not being valued by their managers was another strong feature of the environment. A recent study exploring predictors of job satisfaction among Norwegian nurses identified satisfaction with the local leader as the most important explanatory variable for job satisfaction, with positive evaluation of top management also featuring strongly [30]. Similarly, an exploratory qualitative study of 24 health workers in Viet Nam identified appreciation by managers, colleagues and the community as one of the main motivating factors [31].

The mid-level providers were slightly more positive about their work relationships and the degree of control they have over their practice. There are indications that these workers were not initially well accepted by health staff trained to international levels. For example, in Malawi the government was urged by the nurses and midwifery council to abolish the enrolled nursing programme in the early 1990s and instead to focus on training registered nurses. Training of enrolled and auxiliary nurses was also stopped in Ghana and Zambia. However, Dovlo argues that as these cadres have developed, and as delegation of tasks has been accompanied by delegation of responsibility, "initial hostility changed to fruitful collaboration and to mutual recognition of new professional turfs" [7]. This is less likely to be the case where these cadres are relatively new. Our results also indicate that the mid-level medical cadres report significantly poorer working relationships than the nursing cadres, suggesting that the nursing cadres may be more accepted by work colleagues. As Malawi is a country that has few highly qualified, experienced medical or nursing staff, it is likely that mid-level providers do have a considerable degree of control over their practice, which explains why there is less dissatisfaction with this aspect of the work environment. Indeed, several studies have indicated that the scope of practice has been gradually extended for many mid-level cadres in recent years [32,7].

Strong positive correlations between subscale 1 of the HPWI and the Maslach Burnout Inventory indicate that an inadequately resourced health care environment is associated with emotional exhaustion, as more than one third of respondents scored high on the emotional exhaustion scale. Maslach et al. report mean scores for those working in medicine as emotional exhaustion 22.19 (SD 9.53), depersonalization 7.12 (SD 5.22) and personal accomplishment 36.53 (SD 7.34). Peltzer et al. reported that mean scores for doctors in South Africa are comparable to these, although they report a lower personal accomplishment score 17.4 (SD 6.8). This study reports a higher personal accomplishment mean 35.22 (SD 9.73). However the pattern of one third of the sample scoring high on emotional exhaustion coupled with more than 40% of the sample scoring low on personal accomplishment indicates that burnout may be a problem for many of these cadres. A previous study conducted with nurses in Malawi similarly found burnout to be a problem [33].

A range of correlations highlights the salience of inadequate resources in the work environment to job dissatisfaction, dissatisfaction with one's profession, thinking about leaving one's job and, more worryingly, to mid-level providers' active plans to seek other employment and plans to leave their jobs within the next 12 months. These findings not only confirm the relationship between organizational attributes and job satisfaction and retention that has been found to exist in high-income countries [12-14,16], but also gives a clear indication of the inadequacy of adopting a strategy of training and employing mid-level cadres in the absence of strategies to strengthen and improve other aspects of the health environment in resource-poor settings.

Management support (subscale 2) also correlates with dissatisfaction with current job and actively seeking other employment. Published research generally reports positive statistical relationships between the greater adoption of human resources (HR) practices and business performance [34], yet strategic HR management initiatives are still relatively rare in low-income countries. Manongi et al.'s study of primary health care facilities in Tanzania also found that lack of supervision and feedback left staff feeling unsupported and undervalued.

Working relationships (subscale 3) correlated with emotional exhaustion. Staff experiencing high levels of emotional exhaustion reported significantly poorer working relationships than those categorized as having moderate levels of emotional exhaustion. This indicates the importance of ensuring that mid-level providers are accepted by other health care workers they work alongside. There was also a correlation with degree of satisfaction with profession and with stated likelihood of leaving the job over the next 12 months, suggesting that poor working relationships may be a significant push factor for these cadres.

Autonomy/Control over practice (subscale 4) correlates with degree of satisfaction with salary and with current job assignments, those staff who believe they have less control over their practice indicating that they are less satisfied with salary and current job assignments. These results again highlight the importance of good HR management systems in allowing staff to practise to their full potential.

Medical cadres were significantly less satisfied than nursing cadres with their job and with their current profession, and reported poorer working relationships. These findings are not surprising, given the lack of a career structure for mid-level medical cadres. There is a widespread perception that they are trained to a level at which they are useful, and then abandoned [35]. This lack of a career structure may lead to a feeling of being trapped; such feelings are unlikely to result in good performance [Martineau T, Lehmann U, Matwa P, Kathyola J, Storey K. Factors Affecting Retention of Different Groups of Rural Health Workers in Malawi and Eastern Cape Province. Unpublished report. Geneva: WHO Alliance for Health Policy and Systems Research, 2006]. This cadre of staff has been described as a major resource "who in an unofficially recognised form at the moment provide the backbone of surgery at the district level" [Bowie C: Mid-term review of Surgical Officer Training Programme. Unpublished Report.2007]. Given the recent evidence of the clinical efficacy and cost-effectiveness of members of this cadre, there is a danger that the problems with their training and career structure may be overlooked. Addressing these strong push factors may be critical to retaining this cadre.

## Conclusion

This research has highlighted the importance of motivating the work performance of mid-level providers in low-income countries. It has described areas that must be addressed to create a more motivating work environment, and has demonstrated important differences in the work satisfaction of medical and nursing mid-level providers. We have also identified crucial issues that must be addressed in this regard. Finally, we have delineated the Health Providers' Work Index, based on a previous measure of work environment among nurses, and shown it to be a valuable instrument with a distinct factor structure with predictive value. The Health Providers' Work Index can be used in low-income contexts and with a cadre of health providers for which it was not originally intended. Our findings and this new instrument provide both a motivation and means for further research on improving the performance of new cadres of human resources for health in low-income countries.

## Competing interests

The authors declare that they have no competing interests.

## Authors' contributions

EM participated in the literature review, study design and data collection and drafted this paper. CB participated in the study design and data collection and edited the paper. OM participated in the literature review, study design, data collection and analysis. FM participated in the data collection, data cleaning and preliminary analysis. MM and DH conducted the data analysis and wrote part of the results section of the paper. MC contributed to the data analysis. CN and MM edited the paper.
